# The Mode-of-Action of Targeted Alpha Therapy Radium-223 as an Enabler for Novel Combinations to Treat Patients with Bone Metastasis

**DOI:** 10.3390/ijms20163899

**Published:** 2019-08-10

**Authors:** Mari I. Suominen, Timothy Wilson, Sanna-Maria Käkönen, Arne Scholz

**Affiliations:** 1 Pharmatest Services Ltd., Itäinen Pitkäkatu 4, 20520 Turku, Finland; 2 Aurexel Life Sciences Ltd., Mikonluodontie 55, 21240 Askainen, Finland; 3 Institute of Biomedicine, Faculty of Medicine, University of Turku, 20500 Turku, Finland; 4 Bayer AG, Research and Development, Pharmaceuticals, Müllerstr 178, 13353 Berlin, Germany

**Keywords:** targeted alpha therapy, TAT, bone metastasis, osteoclast, osteoblast, prostate cancer, radium-223

## Abstract

Bone metastasis is a common clinical complication in several cancer types, and it causes a severe reduction in quality of life as well as lowering survival time. Bone metastases proceed through a vicious self-reinforcing cycle that can be osteolytic or osteoblastic in nature. The vicious cycle is characterized by cancer cells residing in bone releasing signal molecules that promote the differentiation of osteoclasts and osteoblasts either directly or indirectly. The increased activity of osteoclasts and osteoblasts then increases bone turnover, which releases growth factors that benefit metastatic cancer cells. In order to improve the prognosis of patients with bone metastases this cycle must be broken. Radium-223 dichloride (radium-223), the first targeted alpha therapy (TAT) approved, is an osteomimetic radionuclide that is incorporated into bone metastases where its high-linear energy transfer alpha radiation disrupts both the activity of bone cells and cancer cells. Therefore, radium-223 treatment has been shown preclinically to directly affect cancer cells in both osteolytic breast cancer and osteoblastic prostate cancer bone metastases as well as to inhibit the differentiation of osteoblasts and osteoclasts. Clinical studies have demonstrated an increase in survival in patients with metastatic castration-resistant prostate cancer. Due to the effectiveness and low toxicity of radium-223, several novel combination treatment strategies are currently eliciting considerable research interest.

## 1. Introduction

Bone metastasis is commonly associated with significant clinical complications which cause severe decrease in quality of life due to pain, skeletal-related events, such as pathologic fractures and spinal cord compression, and reduced patient mobility [1,2]. Furthermore, the presence of bone metastases is associated with a poor prognosis and reduced overall survival in both breast cancer and prostate cancer [3,4,5,6,7]. It is a recurring complication especially in prostate cancer, where bone is the primary site of metastatic disease [8]. In metastatic castration-resistant prostate cancer (mCRPC), bone metastases dominate the clinical picture of the disease, and bone metastasis has been shown to affect up to 90% of patients with mCRPC [8,9,10]. Bone metastasis is also highly frequent in breast, thyroid, and bladder cancer, as well as non-small-cell lung cancer and renal cell carcinoma [11,12]. In metastatic breast cancer, for example, ~71% of patients have been found to develop bone metastases [11]. Additionally, multiple myeloma is clinically characterized by the infiltration of the bone marrow with differentiated plasma cells that cause lytic lesions in large bones and vertebrae.

Bone metastases form as a result of disseminated tumor cells (DTCs) spreading to bone tissue and persisting there until progressing into metastatic growths. The metastatic process is initiated by activation of DTCs during bone remodeling and progresses into a vicious cycle of self-amplification. In the vicious cycle, factors secreted by cancer cells induce the activation of osteoclasts to break down bone and osteoblasts to form excessive new bone. This ultimately leads to a further release of growth factors and, consequently, added tumor growth. Breaking this vicious cycle has been proposed as a central strategy in the treatment of bone metastasis. Bisphosphonates and inhibitors of receptor activator of nuclear factor kappa-Β ligand (RANKL) have been used to prevent bone resorption, but new strategies are also needed to improve the quality of life and life expectancy of patients with cancers involving bone metastases.

Radium-223 belongs to the class of TATs. It receives its targeting properties from being an osteomimetic that homes into bone tissue, not only due to its physical similarity with calcium but also through active incorporation by osteoblasts [13,14]. It emits high-energy alpha particles that cause double-stranded DNA breaks (DSBs) eliciting cell death which is confined to the cancer cells and the surrounding tumor microenvironment due to the short range of the radiation, thus limiting toxic side effects. As an added benefit, the toxic effects of alpha radiation on the tumor microenvironment disrupt the activity of both osteoblasts and osteoclasts helping to break the vicious cycle of metastasis by interrupting the cross talk between cancer cells and osteoblast/osteoclasts [15,16]. New, promising therapy combination strategies are also emerging in conjunction with radium-223 therapy. Radium-223, which acts at a molecular level via the induction of difficult-to-repair, clustered DNA double strand breaks, shows potential of enhanced efficacy in combination with DNA damage repair inhibitors. Immuno-oncology treatments such as checkpoint inhibitors have provided an immense breakthrough in cancer therapy. However, widespread adoption of immune checkpoint therapy to treat cancers is obstructed by the low response rate in some cancer types [17]. In this setting, radium-223 treatment may enhance the activity of immuno-oncological treatments by various mechanisms.

In addition to the use of drug combination strategies to improve antitumor efficacy and increase survival, the use of the bone supportive agents such as denosumab and bisphosphonates, which are approved in mCRPC and other cancers that frequently result in the development of bone metastases, may also help improve the bone health safety profile of treatment strategies containing radium-223 [18,19]. The aim of this review is first, to elucidate the mode of action of radium-223 in breaking the vicious cycle of bone metastasis and second, to discuss how to utilize this unique feature in combination with novel emerging cancer treatments.

## 2. Disseminated Tumor Cells and Dormancy in Bone Metastasis

Bone metastases originate from DTCs that have left the primary tumor and entered the circulation, reaching the bone marrow and persisting in the new environment, with some of them eventually progressing to develop metastases. DTCs are known to harbor stem cell-like properties, such as low proliferative activity, resistance to apoptosis, unlimited ability for self-renewal, and differentiation [20]. The presence of DTCs is an independent factor of poor prognosis [21,22,23], and DTCs are often found in the bone marrow of patients with breast and prostate cancer at a very early phase [21,22,24]. Thus, it is vital to understand what makes tumor cells dormant, what wakes them up and induces their growth into metastases, and how cancer treatments affect this process.

It has been demonstrated that increased bone remodeling increases the number of bone metastases in both breast and prostate cancer mouse models [25,26]. It seems that the prevalence of bone metastases can be explained by the increased activation of the DTCs present, rather than by an increased number of cancer cells homing to bone tissues. Quite interestingly, the longest clinical tumor recurrence times, a decade and more, also coincide with the cycle of renewal of all bone in the skeleton, taking 10–20 years [27], underlining the relevance of remodeling in the formation of metastases.

One of the early events in a new bone remodeling cycle is the formation of new blood vessels in the remodeling site. Angiogenesis is necessary for normal bone remodeling, bone growth, and fracture repair, and it has been demonstrated that while stable endothelium supports tumor dormancy, activated sprouting endothelium in fact induces tumor growth [28]. Thrombospondin 1 binding on the stable endothelium has been described to be crucial for dormancy, whereas transforming growth factor β1 (TGF-β1) and periostin expression induces tumor growth near the sprouting vessels [29]. Accelerated bone turnover by parathyroid hormone (PTH) is also known to induce expansion of the hematopoietic stem cell (HSC) niche [30] which offers a safe haven for dormant tumor cells [31]. Thus, suppressing the level of bone turnover, that is, suppressing the activity of the bone-resorbing osteoclasts and the bone-forming osteoblasts, should be a viable strategy for keeping the DTCs in a dormant state, resulting in the prevention of bone metastases.

## 3. The Tumor Microenvironment in Bone Metastasis

The bone microenvironment is intricately involved in the formation of metastases, being involved in the process even before tumor cells leave the primary tumor. Facilitators of metastasis, including monocytes (macrophages), mesenchymal stem cells, and immature myeloid cells, are expanded in and mobilized from the bone marrow and guided to the primary tumor by cytokines secreted by the tumor [32,33]. Subsequently, through education by tumor cells, these cells become tumor-associated macrophages (TAMs), cancer-associated fibroblasts (CAFs), and myeloid-derived suppressor cells (MDSCs), respectively. These in turn assist in tumor growth and the metastatic process by inducing immune evasion, angiogenesis, epithelial-to-mesenchymal transition, and invasion [34,35]. In bone, the invading tumor cells induce changes in both osteoclasts and osteoblasts as well as their precursors. Depending on the activated cell types, tumor growth in bone results in either an osteolytic (bone resorbing), osteoblastic (bone forming), or a mixed bone lesion. In breast cancer, these lesions are mostly osteolytic (~75%), while the majority of prostate cancer bone metastases are osteoblastic [36]. It should be noted, however, that in most patients both lesion types are present, and at the cellular level, both bone resorption and formation is induced by tumor cells [37].

Bone metastases progress as a result of a vicious cycle of self-amplification, where factors secreted by the cancer cells induce the breakdown of bone or the formation of new bone, leading to a release of growth factors, further supporting tumor growth. Here, the osteolytic and osteoblastic cycles are presented separately for clarity, despite the fact that in a clinical setting both processes often co-exist in the same patient and even in the same metastatic site.

### 3.1. The Vicious Cycle of Osteolytic Bone Metastasis

**Figure 1:**
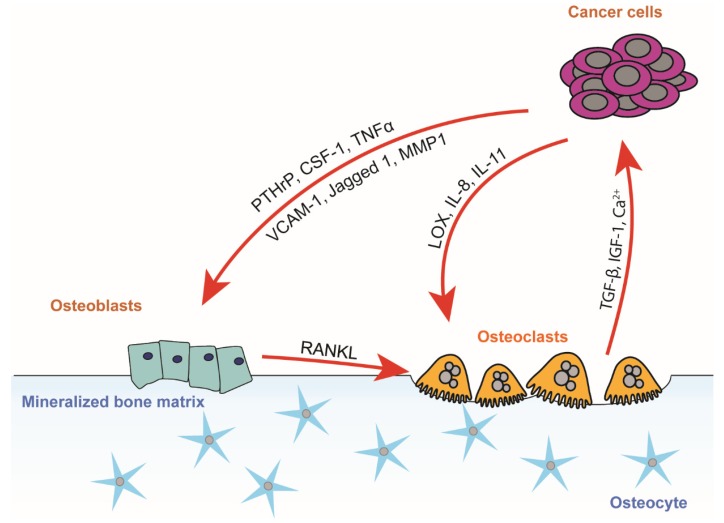
The vicious cycle of osteolytic bone metastasis. In the vicious cycle of osteolytic bone metastasis, cancer cells secrete factors that enhance osteoclast differentiation either directly or indirectly through pre-osteoblasts and osteoblasts. TGF-β, IGF-1, and calcium released by resorbing osteoclasts induce tumor growth and further secretion of osteoclastogenic factors. Abbreviations: PTHrP: parathyroid hormone-related protein, CSF-1: colony stimulating factor 1, TNFα: tumor necrosis factor α, VCAM-1: vascular cell adhesion molecule 1, MMP1: matrix metalloproteinase 1, TGF-β: transforming growth factor β, IGF: insulin-like growth factor, IL-8 and IL-11: interleukins 8 and 11, LOX: lysyl oxidase.

In healthy bone tissue, bone formation and bone resorption are in homeostasis. In osteolytic metastases, resorption and formation become uncoupled due to factors secreted by cancer cells. These factors are able to induce osteoclast formation both directly and indirectly through osteoblasts (Figure 1). Parathyroid hormone related peptide (PTHrP), which leads to increased local bone resorption, and TGF-β1, which is a ubiquitous growth factor in the bone microenvironment, were the first molecular players identified in cancer-induced osteolysis. Breast cancer cells produce and secrete PTHrP, which increases bone resorption indirectly by binding to the PTH receptor in osteoblasts [38]. Osteoblasts in turn secrete less osteoprotegerin (OPG), which reduces bone resorption, as well as more RANKL, which binds to the RANK on osteoclasts and induces differentiation and subsequently bone resorption. This results in an imbalance with excessive resorption-inducing signal molecules compared to formation-inducing molecules. Additional factors secreted by cancer cells, such as hypoxia-induced lysyl oxidase (LOX), colony stimulating factor 1 (CSF-1), tumor necrosis factor α (TNFα), vascular cell adhesion molecule 1 (VCAM-1), matrix metalloproteinase 1 (MMP1), Jagged 1, and interleukins 8 and 11, also induce the activation of osteoclasts, some of them directly, independently of RANK, enhancing the imbalance [27,32,39,40,41].

As the newly activated osteoclasts resorb bone, growth factors such as TGF-β, insulin-like growth factor 1 (IGF-1), and bone morphogenetic proteins (BMPs) that have been deposited in bone are released. They enhance the growth of cancer cells and the subsequent secretion of osteoclast activation factors. In addition to its effects in bone, TGF-β is also important in a plethora of cellular functions that can promote tumor formation, such as epithelial-to-mesenchymal transition, invasion, angiogenesis, and immune tolerance [42]. Moreover, the calcium released from bone as a result of resorption can act as a growth factor in cancer cells that express the calcium-sensing receptor [43,44]. Furthermore, immune cells are known to play an important part in the vicious cycle. Activated T cells express RANKL and are able to induce the differentiation of osteoclasts in vitro without any supporting chemokines [45]. TGF-β in turn inhibits the proliferation of T cells, helping tumor cells to evade their effects [46].

### 3.2. The Vicious Cycle of Osteoblastic Bone Metastasis

**Figure 2:**
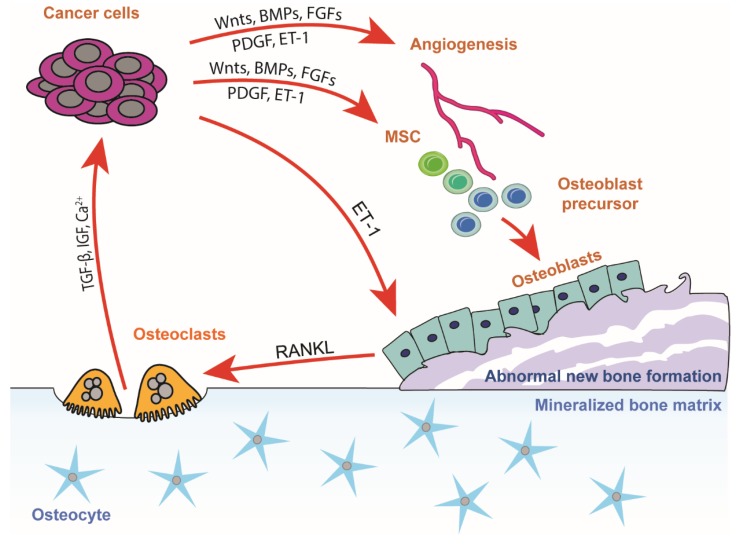
The vicious cycle of osteoblastic bone metastasis. In the vicious cycle of osteoblastic bone metastasis, cancer cells secrete factors that induce angiogenesis and differentiation of MSCs to osteoblasts. Cancer cells utilize the perivascular niches offered by the sprouting endothelium. Osteoblasts induce differentiation and activation of osteoclasts, and growth factors released by bone resorption support the growth of tumor cells. Abbreviations: BMPs: bone morphogenetic proteins, FGFs: fibroblast growth factors, MSC: mesenchymal stem cell, PDGF: platelet-derived growth factor, ET-1: endothelin 1, TGF-β: transforming growth factor β, IGF: insulin-like growth factor.

While most breast cancers are primarily osteolytic, prostate cancers tend to be osteoblastic, forming new, unorganized, and weak bone, or a clear mixture of the two types (Figure 2). One constitutive factor behind the osteoblastic reaction seems to be prostate-specific antigen (PSA). PSA is a serine protease and cleaves many proteins important in bone metabolism, releasing, e.g., IGF-1 and TGF-β2 from their binding proteins to stimulate osteoblasts [47], as well as osteoblast-inactivating PTHrP [48]. Additionally, the induction of PSA through a transgene in a PSA-negative prostate cancer cell line has been shown to shift the bone reaction from osteolytic to osteoblastic [49]. However, in osteoblastic metastasis of prostate cancer, strong osteoclastic resorption is also typical, and bone resorption marker levels in patients with prostate cancer can be even higher than in patients with osteolytic breast cancer [50,51]. Osteoblasts need a resorbed bone surface onto which new bone can be formed and thus require an increased number of bone multicellular units, in addition to increased osteoblast number and activity within one bone multicellular unit, in order to achieve the vast amount of new, pathological bone observed in osteoblastic metastases.

Prostate cancer cells stimulate mesenchymal stem cells (MSCs) toward osteoblastic differentiation by secreting endothelin 1 (ET-1), wingless and Int1 proteins (Wnts), BMPs, TGFs, fibroblast growth factors (FGFs), and platelet-derived growth factors (PDGFs), leading to an increased number of osteoblasts. These factors also induce angiogenesis, expanding the HSC niche that prostate cancer cells can inhabit [27]. Interestingly, ET-1 has been found to be secreted also from breast cancer cells that produce osteoblastic lesions, underlining its overall significance in the osteoblastic lesion type [52].

The osteoblasts, in addition to forming new bone tissue, secrete RANKL, which induces osteoclast differentiation and bone resorption. This, as described above in the context of osteolytic metastasis, releases TGF-β, IGF-1, and BMPs, as well as calcium, which enhances the growth of cancer cells and drives the cycle forward.

## 4. Bone Supportive Agents

### 4.1. Bisphosphonates

Bisphosphonates are pyrophosphate analogs that bind to hydroxyapatite and inhibit bone resorption. Their mode of action is the inhibition of the mevalonate pathway in osteoclasts which disrupts the intracellular enzymatic functions needed for bone resorption [53]. Bisphosphonates are currently used as the main treatment for osteoporosis, and also for patients with bone metastases. Bisphosphonates have very few side-effects and are efficient in decreasing pain and protecting bone, thus preventing skeletal-related events [54]. In combination with radium-223, abiraterone, and prednisone/prednisolone, a lower fracture rate has been observed in patients receiving bisphosphonates or the RANKL inhibitor denosumab [55].

Two large clinical trials, AZURE and ABCSG-12, have been performed to study the efficacy of zoledronic acid in preventing breast cancer bone metastasis. The studies concluded that zoledronic acid increases disease-free survival in postmenopausal women and in women over 40 but not in premenopausal women [56,57]. Interestingly, patients with estrogen receptor-positive (ER+) cancer treated with goserelin, a bone resorption-inducing gonadotropin-releasing hormone agonist, benefitted from zoledronic acid treatment in ABCSG-12 [58], while ER+ patients that were not given goserelin in AZURE did not [56]. Thus, these results suggest that inhibiting increased bone resorption can benefit patients in risk of developing bone metastasis, namely by disrupting the osteolytic metastasis cycle or by preventing cancer cells either from finding refuge in the bone metastatic niche or awakening from dormancy. This is supported by a recent study demonstrating increased distant disease-free survival in postmenopausal patients with breast cancer on aromatase inhibitor therapy with oral osteoporosis treatment [59].

### 4.2. RANKL Inhibitor

The critical importance of the RANK/RANKL/OPG pathway in bone resorption led to the development of the fully human RANKL antibody denosumab, which is approved for the treatment of both osteoporosis and bone support of breast and prostate cancer. It has proven to be very effective in inhibiting bone resorption and preventing skeletal-related events in patients with breast and prostate cancer with bone metastases [60,61]. Similarly, RANKL inhibition has been shown to prevent the occurrence of bone metastases in patients with prostate cancer [62]. When used as an adjuvant supportive treatment in patients with breast cancer receiving aromatase inhibition therapy, denosumab seems to increase time to first fracture as well as improving disease-free survival [63,64]. Thus, the inhibition of the vicious metastasis cycle in bone tissue at a single point, such as RANKL signaling in the case of denosumab, seems to be an effective strategy in reducing the deleterious effects of bone metastases.

## 5. Mechanism of Action and Combination Potential of Radium-223

Radium-223 is a targeted alpha therapy (TAT) compound. TAT refers to a class of radioactive alpha emitting isotopes with short-range, high linear energy transfer (LET) of radiation with the potential of carrying vector conjugates [65,66]. TATs induce complex, difficult to repair DSBs that result in cytotoxicity [67,68], and there are no known resistance mechanisms towards alpha particles. Although TATs are delivered systemically, the short range (less than 100 µm/10 cell diameters) [69] and high LET delivers an end-organ scatter of alpha particles limited to cancer cells and the tumor microenvironment with less damage to surrounding normal tissues outside the alpha emission path range [65,70,71].

The biochemical properties of specific TAT compounds determine the feasibility of conjugation to a vector, as well as determining which of three possible targeting strategies the TAT compound exhibits: 1. self-targeting (physiologic integration by mimicking a natural essential ion); 2. passive targeting (accumulation in tumor areas with leaky vasculature); 3. active-induced targeting (specific ligand-receptor interactions between small molecule/peptide/antibody-labeled radionuclide and target cells) [72].

Radium-223 is a calcium-mimetic isotope that has been shown to incorporate into sites of increased bone turnover and osteoblastic activity due to both physical properties and active incorporation by osteoblasts, making it a self-targeting TAT [13,14,16,70,73]. Compared to beta radiation, for example, alpha radiation has a significantly shorter range and higher linear energy transfer which allows the treatment of metastatic tumors with minimal toxic effects to surrounding noncancerous tissue [74]. The shorter range of radiation results in milder myelotoxicity [13,75], and the higher LET leads to stronger induction of DSBs in tumor cells [76]. Therefore, radium-223 has become an appealing choice for the treatment of bone metastasis. For a summary of the most important nonclinical studies reviewed here, please see Table 1.

Radium-223 is the first-in-class commercially available TAT approved for the treatment of patients with mCRPC with bone metastases. In addition to increasing overall survival in patients with mCRPC, radium-223 increases time to first symptomatic skeletal related event (SSE) and decreases the number of SSEs [85,86]. While radium-223 has shown signs of biological activity in advanced breast cancer patients with bone-dominant disease in early clinical trials [87,88], it remains unclear if this could ultimately translate into a survival advantage.

Preclinical studies have recently demonstrated that radium-223 is indeed incorporated into the bone matrix where it inhibits the proliferation of breast cancer cells and the differentiation of osteoblasts and osteoclasts. In an established osteolytic breast cancer bone metastasis setting, radium-223 prevented tumor-induced cachexia and decreased osteolysis by 56% and tumor growth by 43% [15]. Radium-223 also induced DSBs in cancer cells in vivo and extended survival as a monotherapy and in combination with zoledronic acid. Similar positive results have been demonstrated in the syngeneic 5TGM1 mouse multiple myeloma model, where radium-223 decreased osteolytic lesion area [79], and in the MX-1 osteolytic breast cancer bone metastasis model in rats, where radium-223 increased symptom-free survival [13].

The binding of radium-223 to bone tissue has been demonstrated to occur due to active incorporation by osteoblasts in addition to the passive binding of radium-223 as a calcium mimetic to hydroxyapatite [15]. Additionally, the differentiation of osteoclasts was shown to be inhibited dose-dependently by radium-233. This effect is driven either by direct disruption of osteoclast function by high energy alpha particles emitted by radium-223 deposited near them in areas of active bone remodeling or by inhibiting RANKL secretion by osteoblasts, or the combination of both of these mechanisms. Interestingly, radium-223 has been found to be deposited in the midst of prostate cancer growths in mice xenograft models [16], suggesting a direct effect on tumor metastasis. However, the extent and nature of co-localization with tumor cells remains to be elucidated.

Radium-223 also inhibits the vicious cycle of osteoblastic metastasis. In the LuCaP 58 patient-derived prostate cancer xenograft model, radium-223 was deposited in the intratumoral bone matrix and DNA DSBs were induced in cancer cells within 24 h after treatment [16]. This resulted in the inhibition of tumor-induced osteoblastic bone growth and the preservation of normal bone architecture, leading to reduced bone volume. These effects in tumor-bearing bone occurred through radium-223-induced suppression of abnormal bone metabolic activity as evidenced by a decreased number of osteoblasts and osteoclasts and a reduced level of the bone formation marker PINP. Furthermore, the treatment with radium-223 resulted in lower PSA values and reduced total tissue and tumor areas compared to the vehicle control, indicating that radium-223 constrains prostate cancer growth in bone. Similar findings were also obtained in the LNCaP prostate cancer xenograft model. Preclinical data, however, indicate that radium-223 may need to be combined with other agents to affect larger bone metastasis areas, especially in tumor types with weaker osteoblastic reactions [82].

The mode of action of radium-223 results in a very favorable risk-benefit profile, especially in terms of limited hematotoxicity. After intravenous injection, radium-223 is rapidly cleared from the bloodstream. Thereafter, radium-223 homes into bone tissue where the short range of emitted alpha particles largely limits the effects of radium-223 to the cells of the bone microenvironment, while excess radium-223 is excreted via the gut. In animal studies, no signs of bone marrow toxicity or body weight loss have been observed with therapeutic doses of radium-223 [13], and even high doses have not been shown to inactivate hematopoietic cells [77]. In good agreement with this, the favorable safety profile in clinical trials [89,90] is likely the result of its targeting properties, its mode of excretion, and the short range of the alpha particles, minimizing unwanted radiation to cells beyond the bone microenvironment, the bone marrow cells surrounding bone metastases, and the cells of the gastrointestinal epithelial layer [82,91]. In a retrospective analysis of 1021 patients treated with radium-223 dichloride alone, less than 10% of patients showed one of the most frequently observed adverse events related to radium-223 treatment, such as anemia, thrombocytopenia, fatigue, diarrhea, vomiting, and bone pain. Notably, most of these adverse events were also observed in placebo-treated patients in a randomized phase 3 trial [92]. The risk of hematological side effects may increase in combination with hematotoxic chemotherapeutics [55,93,94]. The potentially negative effects on bone health which may occur especially in certain combination regimens with 2nd generation anti-androgens in patients with disease mediated impairment of general bone health can be diminished by concomitant treatment with bisphosphonates or denosumab [19,55]. Specifically, the combination of radium-223 with abiraterone and prednisone/prednisolone is currently contraindicated in the European Union due to an increased fracture risk. In general, however, combination therapies with radium-223 are broadly feasible in the clinical setting.

### 5.1. Radium-223 Therapy in Combination with DNA Damage Repair Inhibitors

The ability of radium-223 to home into bone tissue and cause DNA DSBs to disrupt the function of both cancer and bone cells has led to considerable interest in using radium-233 in combination with DNA damage repair (DDR) inhibitors. The DDR is the name used collectively for a series of complex signaling pathways that secure the integrity of the genome in eukaryotic cells [95]. This combination is hypothesized to provide clinically relevant synergy in the induction of cancer cell death due to the mode of action of the two therapies—radium-223 treatment induces DSBs in the bone microenvironment which are then not repaired due to DDR treatment. Recent results with the inhibition of ATR (ataxia telangiectasia and Rad3-related) kinase, which is activated by a broad spectrum of DNA damages, including DSBs, in combination with radium-223 treatment have been promising. In the LNCaP intratibially injected prostate cancer model mimicking CRPC with bone metastases, combination treatment with radium-223 and an ATR inhibitor increased intratumoral levels of DNA damage and decreased total tumor lesion area and PSA values suggesting the potential of disrupting the vicious cycle of bone metastasis [80]. A recent in vitro study has additionally shown that ATR inhibition potentiates the inhibitory effect of radium-223 radiation on cell survival [83]. Radium-223 treatment has also been used together with the inhibition of poly (ADP-ribose) polymerase (PARP), which has been shown as monotherapy to be an effective strategy to treat cancers with certain types of DNA repair deficiencies [96]. This concept is further supported by an increase in antitumor efficacy observed when combining either ATR or PARP inhibition with actively-targeted TAT in the form of a mesothelin (MSLN)-targeted thorium conjugate (MSLN-TTC) [81]. Currently, a combination treatment with radium-223 and niraparib, an orally active small molecule PARP inhibitor, is also under evaluation in patients with CRPC with bone metastases (NCT03076203).

### 5.2. Radium-223 Therapy in Combination with Immuno-Oncological Treatments

Radium-223 may also provide benefits when combined with immuno-oncological treatments. A recent preclinical study has demonstrated that radium-223 treatment enhances the sensitivity of various carcinomas to lysis by antigen-specific cytotoxic T lymphocytes through calreticulin-mediated immunogenic modulation [78]. This mode of action may well also inhibit the formation of bone metastases. Radiotherapy in general has been demonstrated to induce the release of danger-associated molecular patterns (DAMPs), such as CRT, HMGB_1_, and secreted ATP, which elicit antigen-presenting cells to activate CD8^+^ T cells to induce immunogenic cell death [97,98]. Radiotherapy is also known to upregulate major histocompatibility complex class I (MHC-I) expression [99,100], which is commonly downregulated in cancerous tissue [101] and associated with poor survival [102,103]. The increase in MHC-I expression together with novel proteins produced by immune cells in response to radiation [104] ultimately leads to the expansion of the T cell repertoire. Actively-targeted TAT in the form of mesothelin (MSLN)-targeted thorium conjugate MSLN-TTC has also been shown to induce immunogenic cell death and secretion of pro-inflammatory cytokines in vitro [84]. In addition to directly enhancing the function of the immune system, osteomimetic radionuclides, such as radium-223, target osteoclasts, which have recently been shown to have immunosuppressive properties [105]. With this in mind, the safety and tolerability of the immune checkpoint inhibitor atezolizumab, a fully humanized and monoclonal PD-L1 antibody, is currently being studied in combination with radium-223 in patients with mCRPC which has progressed following treatment with an androgen pathway inhibitor (NCT02814669). The combination of radium-223 treatment with immune checkpoint blockade may also give rise to additional benefits outside the tumor microenvironment, as recent reports have called attention to the abscopal effect of combining immune checkpoint inhibition and irradiation, that is, the occurrence of systemic antitumor effects resulting in the regression of tumors outside of the irradiated area [106]. As PSA appears to be a constitutive factor in the osteoblastic reaction in bone metastatic prostate cancer, this abscopal effect may bring an added benefit to breaking the vicious cycle of metastasis.

## 6. Conclusions

Targeted alpha therapy with radium-223 provides a two-pronged approach to disrupting the vicious cycle of bone metastasis regardless of its type or tissue of origin, according to preclinical studies. Due to its bone-homing characteristics, radium-223 disrupts not only the activity of cancer cells by eliciting cancer cell death through DNA DSBs, but also the function of both osteoblasts and osteoclasts, thereby simultaneously breaking at least two links of the metastasis cycle.

The targeted nature of radium-223 and its short alpha radiation range cause relatively few side effects, providing a strong rationale for its use in combination treatments. Accordingly, studies related to combining radium-223 treatment with therapies of various modes of action, such as DNA damage repair inhibitors and immune checkpoint inhibitors, are currently under way. These therapy combinations have the potential to significantly improve on the current effectiveness of the treatment of bone metastatic CRPC and bone metastasis from additional tumor types.

## Figures and Tables

**Table 1:** The main findings of radium-223 in nonclinical cancer studies.

Authors	Year	Nonclinical Model	Main Findings	Ref
Henriksen et al.	2002	MT-1 human breast cancer bone metastasis model in rats	Radium-223 increases symptom-free survival.	[13]
Larsen et al.	2006	BALB/c mice	Doses ten-fold higher than therapeutic doses do not cause complete bone marrow suppression.	[77]
Suominen et al.	2013	MDA-MB-231 human breast cancer bone metastasis model in mice	Radium-223 prevents tumor-induced cachexia, decreases osteolysis, induces DNA DSBs, and extends survival alone and in combination with zoledronic acid or doxorubicin.	[15]
Malamas et al.	2016	In vitro	Radium-223 significantly enhances T cell-mediated lysis of prostate, breast, and lung carcinoma tumor cells by CD8^+^ cytotoxic T lymphocytes.	[78]
Suominen et al.	2017	5TGM1 mouse myeloma model	Combination of radium-223 with bortezomib could constitute a novel, effective therapy for multiple myeloma.	[79]
Suominen et al.	2017	LNCaP, LuCaP 58 prostate cancer bone growth models in mice	Radium-223 inhibits tumor growth, tumor-induced osteoblastic bone growth, and protects normal bone architecture. Radium-223 shows a preferential uptake in bone lesions compared to normal bone and deposits in newly formed intratumoral bone matrix. Radium-223 induces DBS in local tumor cells, OBs and OCs.	[16]
Wengner et al.	2018	LNCaP human prostate cancer bone growth model in mice	Treatment with ATR inhibitor BAY 1895344 and radium-223 exhibits synergistic antitumor activity.	[80]
Wickstroem et al.	2018	OVCAR-3 ovarian cancer model in mice	MSLN-TTC increases the antitumor efficacy of ATR and PARP inhibitors.	[81]
Dondossola et al.	2019	PC3 and C4–2B human PCa cell lines in mouse bones	Micro-tumors showed good response to radium-223. Larger tumor areas were not as efficiently controlled by radium-223.	[82]
Bannik et al.	2019	In vitro	Synergistic in vitro effects were observed when radium-223 was combined with the ATR inhibitor BAY 1895344.	[83]
Hagemann et al.	2019	In vitro	MSLN-TTC is able to induce immunogenic cell death and secretion of pro-inflammatory cytokines in vitro.	[84]
